# Measurement of vertical anterior teeth display and lip position at smiling in Kurdish population. Age and gender-based evaluation

**DOI:** 10.1016/j.heliyon.2023.e19465

**Published:** 2023-08-28

**Authors:** Neda AL-Kaisy

**Affiliations:** Department of Prosthetic Dentistry, College of Dentistry, University of Sulaimani, Sulaimani, Iraq

**Keywords:** Anterior teeth display, Lip position, Posed smile, Spontaneous smile

## Abstract

**Statement of Problem:**

As part of the overall facial analysis, smile analysis is an essential component of diagnosis and treatment planning in the esthetic rehabilitation of a patient with missing anterior teeth.

**Purpose:**

The aim of this study was to investigate the effect of age and gender on the amount of maxillary anterior teeth and associated lip position during smiling in the Kurdish population to establish guidelines for rehabilitating edentulous patients.

**Material and methods:**

Video equipment was used to capture images of 80 Kurdish subjects divided into two groups by age and sex: Forty young subjects (20 women and 20 men aged 21–24 years), and another forty old subjects (20 women and 20 men aged 45–65 years). Three frames for each subject were selected: one representing the entire length of maxillary anterior teeth, another the posed smile frame and the third representing the spontaneous widest smile. These images were used to quantify a dentogingival exposure for each anterior tooth on either side using standardized measurement techniques. The Kruskal-Wallis test was used to evaluate and compare differences in teeth and gingival display and the Chi-square test was used to explore the frequency of smile line types (α = 0.05).

**Results:**

There were no significant age or sex differences in the anterior teeth display of posed smile. However, women displayed more maxillary anterior teeth in both age groups. The highest display was for lateral incisors, followed by central incisors and canine (61.3%, 58.9%, and 49.05% in the young group vs 62.05%, 54.5%, and 53.3% in the old group). On the other hand, a significant age difference was observed in dentogingival display of maxillary anterior teeth during a spontaneous smile, including mainly the lateral incisors length with their overlying gingiva (98.5%, 1.46 mm in young vs 92.1%, 0.47 mm in old). Women show insignificant excess gingival display than men. Low smile line (class IV) was the predominant type of smile in posed smiles (60%–62.5%). While the average smile line (class III) was the dominant type of young (52.5%) and the high (class II) of old (40%) in spontaneous smiles.

**Conclusions:**

Age influences the dental and gingival display of anterior teeth in spontaneous smiles but not in posed smiles. Women generally show more gingiva and teeth in all the parameters evaluated than men. The predominant type of smile changed from (class IV) in posed smile to (class III) of young and to (class II) of old subjects in a spontaneous smile. Dental treatments should be individually planned according to age-related dynamic norms.

## 1 Introduction

The anterior teeth are essential in esthetics, phonetics, and food incision. In particular, esthetics is often the primary focus during anterior teeth restoration or replacement. Restoring all of the missing maxillary anterior teeth mandates the dentist to consider the patient's age, gender, race, and facial structure proportions to build normal teeth contour and display in relation to lip position at smiling as close as possible to that obtained with natural teeth of a similar group [1,2]. Esthetic outcome is also critical for patient satisfaction and Oral health-related quality of life. General guidelines are required to aid clinicians in optimizing dentofacial esthetics while satisfying other treatment goals [3].

The smile line (based on the amount of vertical tooth exposure) is a good tool for the assessment of the esthetic appearance of a smile and can be applied universally [4,5]. In complete denture wearers, a smile line exposing the cervical third of the maxillary anterior teeth was considered the most attractive smile [6]. Clinicians are encouraged to create or restore a pleasant facial appearance by developing a balanced and pleasant smile. Therefore, to get a convincing esthetic analysis, both posed and spontaneous smiles must be defined and considered. Spontaneous smiling should be the logical focal point for the esthetic diagnosis of lip tooth relation-ship during smiling [7].

Liébart et al. [8] established four types of smile lines: Class I, with a very high smile line (more than 2 mm gingival display); Class II, with a high smile line (0–2 mm gingival display); Class III, with an average smile line (display of gingival embrasures only); and Class IV, with a low smile line (gingival embrasures and enamel-cement junction not visible).

Four factors influence tooth exposure (lip length, age, race, and sex) [9,10]. The amount of maxillary tooth displayed is inversely proportional to increasing age due to the reduction in tonicity of the orofacial muscles, and loss of elasticity of the upper lip accounts for less maxillary and more mandibular incisor tooth display [10]. Moreover, the incidence of tooth wear is more prevalent in the age group above 50 years, and it is more frequent among males when compared to females [11]. It is also revealed that tooth exposure decreases as lip length increases regarding age and sex [12]. Gender differences affect maxillary teeth display during smiling; women display more maxillary gingivae than men [[13], [14], [15], [16], [17]].

Furthermore, ethnicity could give some characteristic smiling line features. African Americans displayed significantly more gingival tissues than other races or national origins (white American, Chinese, Hispanic, Japanese, and Korean) [16]. Similar findings were described in Asian females who present with a higher smile line than Germanic and Roman females [14]. The smiles have also been found to differ according to culture. Most Western countries are characterized by a big posed smile, whereas in many Eastern countries, like China, a smile without the teeth exposed is traditionally preferred [18].

Various techniques have been used to assess smile esthetics; direct measurement with rulers or calipers, conventional photography, video recording, laser scanning, and stereo photogrammetry. Direct measurement is a simple method but may be susceptible to errors from inadvertent distortion of the soft tissues [9,19]. Previous studies depended on static photographic analysis with or without facial reference markers [20,21]. While numerous studies were performed using digital image manipulation [22,23]. Many other researchers improved the method of esthetic assessment and used videography and software to record a smile rather than a static picture [3,7,13,[24], [25], [26], [27]]. A spontaneous smile is difficult to obtain with static photographs [28], and it is impossible to capture the highest smile line of a patient in a single photographic image. Recently, due to the improvements in video and photographic quality in modern smartphones and the simplicity of their application, protocols have been developed to use them by clinicians to conduct their studies instead of expensive and bulky photographic equipment [13,29].

This study aims to evaluate the relationship between the anterior maxillary teeth’ vertical position and the upper lip smile line in dynamic smiling in the dentate Kurdish population and help clinicians by establishing guidelines for rehabilitating edentulous Kurdish patients. The null hypothesis tested was that no difference would be found in maxillary anterior teeth display and position of smile line in posed and spontaneous smiles between women and men, nor between young and aged groups.

## 2 Clinical implications

In order to accomplish optimal dental esthetic results when treating prosthodontic patients, it is of paramount importance for the clinician to follow established esthetic guidelines. These guidelines mainly related to the maxillary anterior position in relation to the upper smile line during both posed and spontaneous smiling.

## 3 Material and methods

The sample used in this study consisted of 80 healthy Kurdish subjects, 40 young subjects (20 women and 20 men aged 21–24 years), and another 40 old subjects (20 women and 20 men aged 45–65 years). The sample size was calculated using G × Power (V. 3.1.9.7) based on the values of this research pilot study rather than previous studies, as ethnicity and culture could affect some smiling characteristic features. The means and standard deviation of the right central incisor display during the posed and spontaneous smile of twenty subjects (10 young and 10 old subjects) were used. The selection of maxillary central incisors is of utmost importance as these are the key determinant in evaluating smile esthetics [30]. The power analysis showed a total sample size estimate of 22 subjects for each age group in a posed smile (the younger group mean was 6.002 ± 1.92, and the older group mean was 4.68 ± 1.45), and at least 23 subjects for each age group in a spontaneous smile (the young group means 8.75 ± 1.31; and the old group means 7.65 ± 1.58). This is based on the profession at a conventional α level (0.05) and desired power (1– β) of 0.8. Notably, this sample size number was later increased to 40.

The subjects were students, faculty staff, and patients at the College of Dentistry/University of Sulaimani. All subjects were required to have permanent, well-aligned natural maxillary and mandibular teeth up to and including the first molar. No subjects were included in the study if they were missing anterior teeth, visible periodontal disease, anterior restorations, fixed anterior dental prosthesis, excessive dental attrition, a history of orthodontics treatment, gross facial asymmetry, lip irregularities, or a history of lip surgery.

This study did not evaluate smile esthetics but considered a randomly selected natural smile. Research aims were explained, and the subjects signed a consent agreement. The medical ethics committee of the Faculty of Dentistry, University of Sulaimani, approved this study (No:10 on September 01, 2021).

**Fig. 1:**
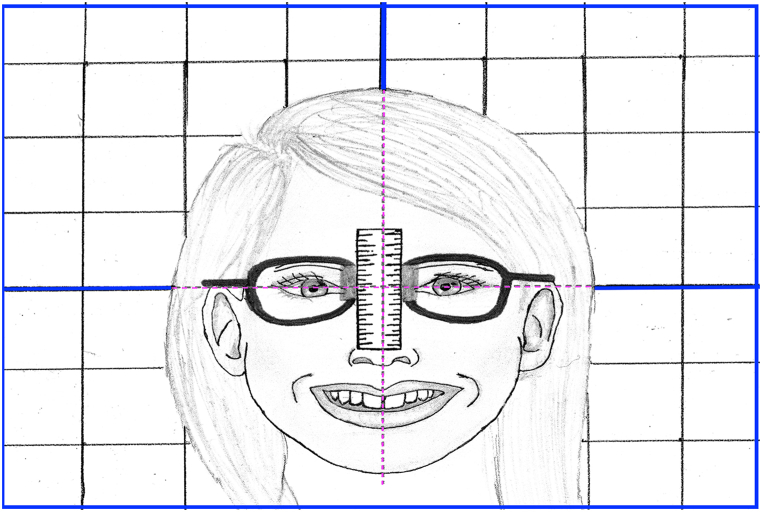
Subject's head position adjustment in relation with longitudinally and transversely striped board fixed behind the subject's head wearing eyeglass frame holding metal ruler by magnet.

A digital video recording was taken for each subject. The video clips were made with a smartphone's 8-MP camera (iPhone 6 iSight; Apple Inc) with a 60-mm (58-mm) Moment lens (Moment Inc([13] fixed to an extendable tripod stand. The stand was adjusted to the subject's mouth level at a 70 cm distance under standardized lighting conditions. Each subject was seated in an upright position on a back-supported and vertically adjustable chair, looking straight ahead. A longitudinally and transversely striped board was fixed on the wall behind the subject's head. The subject's head was adjusted so that the board's vertical midline was bisecting the subject's face, while the horizontal midline passed between the centers of the pupils of the eye (Fig. 1). For anterior-posterior adjustment of the subject's head, the ala-tragus line was adjusted to be parallel to the floor.

The subjects were asked to wear an eyeglass frame holding a metal millimeter ruler to enable calibration in a digital measurement program. The ruler was attached to the glasses vertically by a magnet to facilitate its adjustment (Fig. 1). Before starting a video recording, subjects were given instructions to match the author's performance and encouraged to practice these twice at a minimum. Initially, subjects were asked to reflect their upper and lower lips and expose the whole maxillary anterior teeth to measure its entire length. The next task involved performing a natural posed smile and then relaxing (posed smile is considered when a minimum change in commercial angle to upward movement and slight modifications in eye response without rhythmical mouthing movements or contraction of other facial muscles). Third, subjects repeated the aforementioned smile twice and relaxed. Finally, subjects were asked to perform a full spontaneous smile. One frame time was selected to register and measure the two types of smiles primarily because the repeated continuous posed (unstrained) and spontaneous (strained) smiles enhanced the subject ability to express a true smile representation without hesitation. As a result, subjects could understand the difference in performing each smile which improved their ability to define the specific character for each performance.

One operator (N.M.J) recorded the videos and carried out the measurements for all 80 subjects. Before adjusting the setting of the video clip capturing procedure, N.M.J was overseen by a professional photographer (Y.M.S) to standardize and thus ensure the quality of the video clips and the resulting image files.

The author downloaded each video clip to a secure laptop (MacBook Pro) to protect subjects' privacy and processed the clips using the ‘Imovie Library’ to review the dynamic smiling process. The video clip was stopped at the exact position of each requested smile (natural posed smile and the most dentogingival smile display) and a snapshot was taken. Five images were captured for each subject: one for the entire vertical length of maxillary anterior teeth, three posed smiles and one full spontaneous smile.

**Fig. 2:**
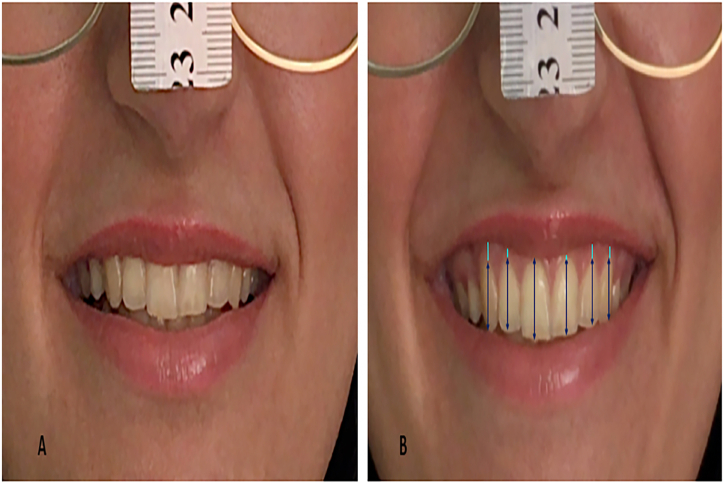
Digital image from videoclip capturing (A) posed smile, (B) spontaneous smile of young subject. Black arrow: measurement of displayed tooth length; blow line: measurement of gingival display over each anterior tooth.

As discussed, subjects were asked to perform a simple posed smile and then relax. At the point of starting the relaxation, a snapshot was taken to represent a posed smile; a process that was repeated three times. For standardization of posed smiles, subjective and objective evaluations were conducted. The measurement of the width of the three posed smile photos was used for objective evaluation as the measured smile width should be approximately identical. To avoid bias in ascertaining the representation of the posed smile from each subject's three resultant images, two other experienced specialist dentists (B.T.K and J.F.A) at Sulaimani College of Dentistry, who are interested in esthetic dentistry, were consulted to make a subjective decision. The three images were viewed together, assessing the entire range of the smile with the whole face, facial muscles, and eye expressions; making them significantly more informative than cropped images. A decision was made to select one photo which subjectively appeared most natural and represented the subject's posed smile to be used for the study measurements (Fig. 2A). The full spontaneous smile (Fig. 2B) was detected as the commercial angle reached the most expansive and highest level, just before starting to relax and returning to the normal relaxed position.

All images were cropped to leave a proportionate area around the lip (to eliminate other facial morphological characteristics), with part of the ruler appearing to help convert the scale from pixel to actual measurements (mm) in the image analysis program (Fig. 2). Image J software [31] was used to conduct the planned measurements (the displays of anterior teeth and gingivae).

The entire clinical crowns’ lengths were measured from the first frame. This represented the vertical distance from the midpoint cervical line to the most incisal edge. In smiling frames, some anterior teeth were covered by either upper or lower lips or both. The displayed crown length of each tooth was measured as a straight line from the middle incisal edge (or tip in case of canine) to the lower border of upper lip. The same operator (N.M.J) recorded all measurements twice and then took an average value. To avoid anatomical variation due to physical size differences between the young and old individuals and to quantify the differences between posed and spontaneous smiling, the dynamic measurements were expressed as a percentage value from the corresponding total tooth length distance [7,17,32] according to the following equation:

$$\left[The\;displayed\;\%\;of\;the\;tooth=\frac{The\;displayed\;amount\;of\;the\;tooth\;\left(mm\right)\times 100}{The\;original\;full\;crown\;length\;\left(mm\right)}\right]$$

Some parts of the anterior teeth were invisible during smiles; either the incisor edges (or the tip of the canines) and/or the cervical tooth part. When the incisal edge (or tip of the canine) was covered by the lower lip while displaying the cervical part during smiling, this invisible part = [original full crown length (minus) displayed crown length]. However, when both parts (the incisal and the cervical) were hidden, each part was calculated separately.

**Fig. 3:**
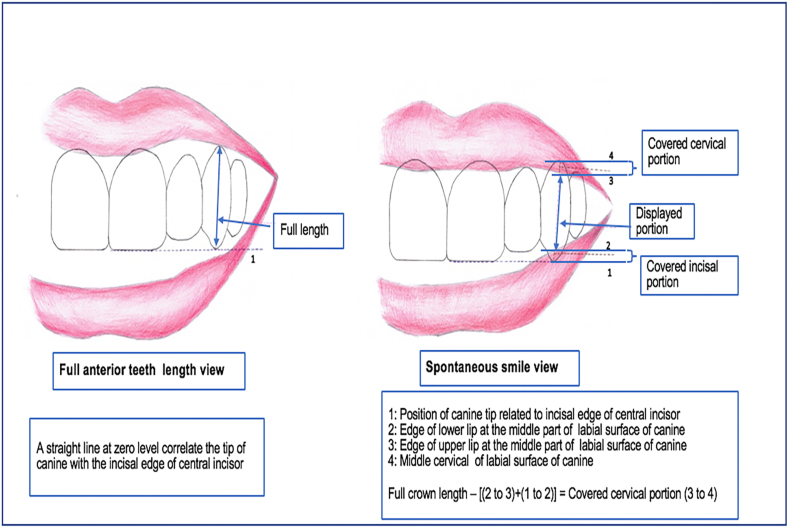
Sketch diagram showing digital measurements of displayed and covered tooth portions.

For example, when a canine was cervically covered by the upper lip and incisally by the lower lip during spontaneous smiling, exposing just the middle part of the tooth, as illustrated in Fig. 3, the measurement of the un-displayed incisal (tip) part was conducted as follow: First from the full teeth length image, the canine tip position is determined by drawing a straight line connecting this tip with any equal level of displayed incisal edge of the adjacent central (or lateral) incisor (Fig. 3). Then, this tip position could be pointed on the smiling view in the same manner as was demonstrated in the image of the full-length teeth. A measurement from this estimated canine tip point to the upper border of the lower lip that covers the canine tip was considered the length of the un-displayed tip portion. Consequently, the covered cervical portion will = full crown length- (displayed canine length + un-displayed incisal portion) (Fig. 3). The resulting displayed and un-displayed teeth values were converted to percentages.

Data were analyzed with statistical software (PASW Statistics for Windows v23; SPSS Inc). A non-parametric test for independent variables, the Kruskal Wallis one way analysis of variance was used to evaluate and compare differences in teeth and gingival display during smiling among all tested groups. Mann Whitney test was used to compare the young and old groups. Chi square test tested the frequency of smile line types (α = 0.05).

## 4 Results

**Fig. 4:**
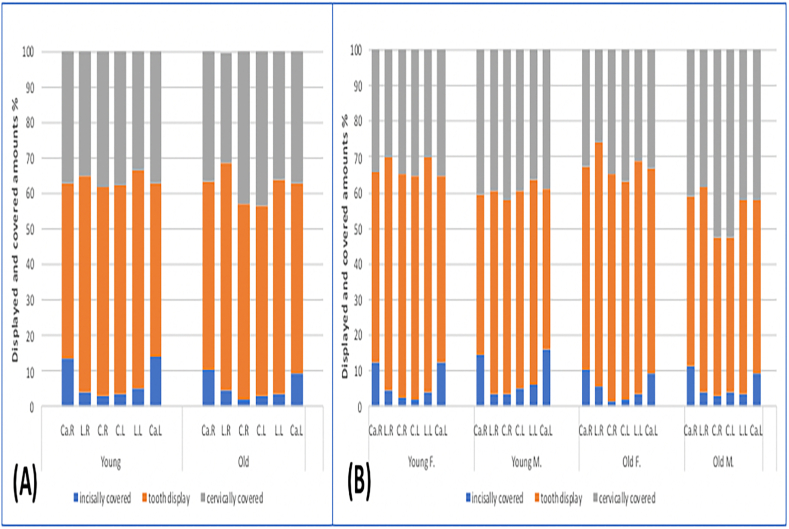
Displayed and covered amounts of maxillary anterior teeth (%) during posed smile. A: comparison between young and old group. B: comparison among all tested groups. C, central incisor; L, lateral incisor; Ca, canine; R, right side; L, left side; F, females; M, males.

The percentage of displayed and un displayed dentogingival portion during posed smile for the six maxillary anterior teeth is presented in Fig. 4. In both age groups (young and old), about 50%–60% of maxillary anterior teeth length was displayed. The most frequently visible tooth in young and old groups was the lateral incisor (61% vs 63.9%, *P* = .339 for the right side, and 61.6% vs 60.2%, *P* = .840 for the left side). On the other hand, the lowest display percentage was for canines, followed by central incisors in young and old age groups (49.4% vs 53%, *P* = .399 for right canine 48.7% vs 53.6%, *P* = .254 for left canine and 58.6% vs 55.3%, *P* = .644 for right central incisors and 59.2% vs 53.7%, *P* = .245 for left central incisors) (Table 1).

**Table 1:** Displayed and covered amount of maxillary anterior teeth (%) at right and left sides during posed smile.

	Groups	Sex	Right side						Left side					
			Ca.R	Mean	La.R	Mean	C.R	Mean	C.L	Mean	La.L	Mean	Ca.L	Mean
**Amount of tooth display %**	**Young**	F	53.6	49.4	65.3	61	62.7	58.6	62.9	59.2	65.8	61.6	52.3	48.7
		M	45.3		56.7		54.5		55.5		57.4		45.1	
	**Old**	F	56.8	53	68.7	63.9	63.4	55.3	61.6	53.7	64.9	60.2	57.5	53.6
		M	47.9		57.5		44.4		43.2		54		48.5	
**Amount of tooth covered cervically %**	**Young**	F	34	37.1	29.9	34.7	34.7	38.3	35.1	37.3	30	33.3	35.3	37.1
		M	40.3		39.5		41.9		39.5		36.6		38.9	
	**Old**	F	32.9	36.3	25.7	31.2	35	42.6	36.6	43.4	31.3	36	33.1	36.9
		M	40.9		38.6		52.8		52.5		42.3		41.9	
**Amount of tooth covered Incisally** **%**	**Young**	F	12.4	13.4	4.8	4.3	2.6	3.1	2	3.5	4.1	5.1	12.4	14.2
		M	14.5		3.8		3.6		5		6		16.1	
	**Old**	F	10.3	10.7	5.5	4.8	1.7	2.2	1.8	3	3.7	3.7	9.4	9.5
		M	11.2		3.9		2.8		4.2		3.7		9.6	

F, females; M, males; Ca, canine; La, lateral incisor; C, central incisor; R, right side; L, left side.

The older subjects' upper lips covered slightly more cervical portions of central incisors. In contrast, the lower lip covered less incisal portion of maxillary anterior teeth than in the young group (Fig. 4A). The differences in the displayed and un displayed parts of anterior teeth between both age groups were not statistically significant (*P* > .05). However, women in both age groups showed more maxillary anterior teeth display than men (Fig. 4B). Older men appeared with the least central incisors displayed percentage than the other tested groups (right and left central incisors for old men 44.4%, 43.2% vs old women 63.4%, 61.6%, vs young women 62.7%, 62.9%, vs young men 54.5%, 55.5%) (Table 1).

**Fig. 5:**
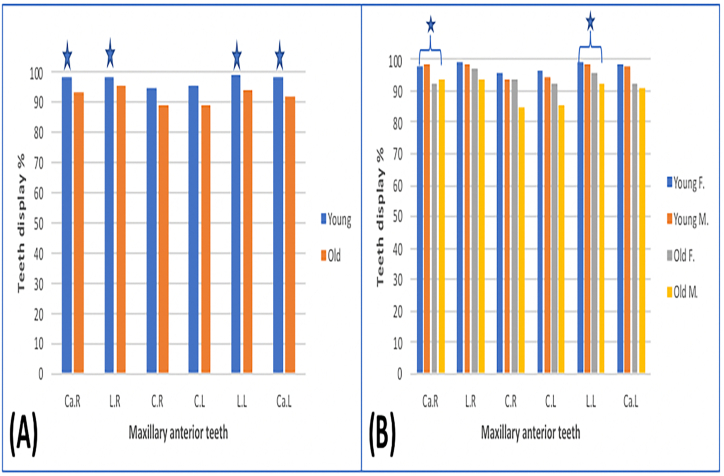
Average amount of maxillary anterior teeth display during spontaneous smile (%). A: comparison between young and old group. B: comparison among all tested groups. C, central incisor; L, lateral incisor; Ca, canine; R, right side; L, left side; F, females; M, males. *Significantly different at *P* < .05.

During spontaneous smile, the mean amount for all maxillary anterior teeth displayed ranged between (82.4%–99.5%). Lateral incisors showed the highest display in both sexes and age groups, while the central incisors were the lowest (Fig. 5A & B). The visible crown height decreased with increasing age and was significantly reduced for lateral incisors (98% vs. 92.2%, *P˂.001* for the right side, and 98.1% vs 92%, *P* = .029 for the left side) and canines (96.5% vs. 90.8% for the right side, *P˂.05*, and 97.1% vs 90.4%, *P˂.001 for* the left side) but not for the central incisors (93.2% vs 86.4%, *P* = .258 for the right side, and 94.4% vs 86.4%, *P* = .149 for the left side) (Table 2 and Fig. 5A). Generally, older men appeared with the least central incisors displayed than the other tested groups and women in both age groups showed more visible crown height than men. However, the differences were not significant. The significant difference was only seen between young men and older women in the right canine and left lateral incisor (Fig. 5B).

**Table 2:** Displayed and covered amount of maxillary anterior teeth (%) at right and left sides during spontaneous smile.

	Groups	Sex	Right side						Left side					
			Ca.R	Mean	La.R	Mean	C.R	Mean	C.L	Mean	La.L	Mean	Ca.L	Mean
**Amount of tooth display %**	**Young**	F	94.8	96.5	96.7	98	92.9	93.2	94.4	94.4	97.4	98.1	95.9	97.1
		M	98.3		99.5		93.6		94.5		98.8		98.5	
	**Old**	F	89.3	90.8	93.5	92.2	90.4	86.4	89.9	86.4	94	92	90.2	90.4
		M	92.2		91		82.4		82.8		90		90.5	
**Amount of tooth covered cervically %**	**Young**	F	3.5	2.2	3.3	1.9	7.1	6.8	5.6	5.6	2.6	1.9	2.12	1.6
		M	1		0.5		6.4		5.5		1.2		1	
	**Old**	F	7.7	7.7	6.5	7.8	9.6	13.6	10.1	13.7	6	8	7.7	8.6
		M	7.8		9		17.6		17.2		10		9.5	
**Amount of tooth covered Incisally** **%**	**Young**	F	1.7	1.3	0	0	0	0	0	0	0	0	2	1.5
		M	1		0		0		0		0		1	
	**Old**	F	3	1.5	0	0	0	0	0	0	0	0	3	1.5
		M	0		0		0		0		0		0	

F, females; M, males; Ca, canine; La, lateral incisor; C, central incisor; R, right side; L, left side.

**Fig. 6:**
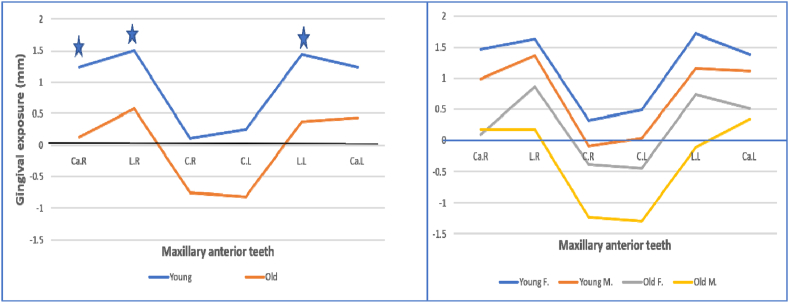
Average amount of gingival display of maxillary anterior teeth during spontaneous smile (mm). A: comparison between young and old group. B: comparison among all tested groups. C, central incisor; L, lateral incisor; Ca, canine; R, right side; L, left side; F, females; M, males. *Significantly different at *P* < .05.

With increasing age, the gingiva was less displayed above all anterior maxillary teeth during the spontaneous smile. It was significantly less above laterals (0.57 mm vs 1.5 mm, *P* = .047 for the right side, and 0.37 mm vs 1.43 mm, *P* = .042 for the left side) and right canine (0.13 mm vs 1.22 mm, *P* = .037). The upper lip covered more cervical portion of the old group central incisors than the young group; it turned gingival display to a negative value (from +0.11 mm to −0.74 mm for the right side and from +0.25 mm to −0.81 mm for the left side). Smile line heights during spontaneous smiling in the old group were reduced by (0.8 mm–1.09 mm) more than in the young group (Fig. 6A). Women have more anterior teeth and gingival visibility compared to men during spontaneous smiling in both age groups. However, the differences were not significantly different (Fig. 6B). The largest gingival display was seen over the laterals, while the least was over the central incisors in all tested groups (Fig. 6A–B).

The prevalence values of various types of smile lines height are shown in Table 3. During posed smiles, the prevalence was statistically different from spontaneous smiles only for young women (*P˂.001*). In posed smiles, 35%–37.5% of both age groups presented an average smile line (class III), and 60%–62.5% had a low smile type (class IV). Young women and men posed smiles presented with an equal percentage of gingival display (40%) (class II, III). Older women smile more intensely and show more gingiva than older men (50% vs. 25%). Three-quarters of older men presented with a low smile line (75%).

**Table 3:** Prevalence of type of smile (%) with respect to age and sexes with posed and spontaneous smiles.

	Posed smile						Spontaneous smile					
	Young group	Old group	*Young women	Young men	Old women	Old men	Young group	Old group	*Young women	Young men	Old women	Old men
	N (%)	N (%)	N (%)	N (%)	N (%)	N (%)	N (%)	N (%)	N (%)	N (%)	N (%)	N (%)
**Class I**	0 (0)	0 (0)	0 (0)	0 (0)	0 (0)	0 (0)	6 (15)	3 (7.5)	5 (25)	1 (5)	1 (5)	2 (10)
**Class II**	2 (5)	0 (0)	1 (5)	1 (5)	0 (0)	0 (0)	11 (27.5)	16 (40)	6 (30)	5 (25)	9 (45)	7 (35)
**Class III**	14 (35)	15 (37.5)	7 (35)	7 (35)	10 (50)	5 (25)	21 (52.5)	13 (32.5)	7 (35)	14 (70)	6 (30)	7 (35)
**Class IV**	24 (60)	25 (62.5)	12 (60)	12 (60)	10 (50)	15 (75)	2 (5)	8 (20)	2 (10)	0 (0)	4 (20)	4 (20)
**Total sample**	40 (100)	40 (100)	20 (100)	20 (100)	20 (100)	20 (100)	40 (100)	40 (100)	20 (100)	20 (100)	20 (100)	20 (100)

Statistically significant differences between the prevalence of posed and spontaneous smile of young women indicated with asterisk (*P*˂.001).

While for spontaneous smiles, a higher percentage of subjects presented with exposed interdental papilla and attached gingiva (class I, II, and III) in both age groups (95% for young and 80% for old). Older women and men presented the same percentage of gingival display (80%), and 20% of each had low smile lines. However, women in both age groups have higher smile lines (class I and II) than men (55% vs 30% in young and 50% vs 45% in old).

## 5 Discussion

Evaluating the dentogingival exposure using both posed and spontaneous smiling should be the focal point for the esthetic diagnosis of lip tooth relationship during smiling [7]. The profound changes from natural aging on the relations between soft tissue and dentition with gender differences should be included in prosthodontics treatment planning. Hence, the present study was conducted to investigate the averages and ranges of these elements in dentate Kurdish subjects to establish more objective guidelines for edentulous patients.

Restorative dentists have generally overlooked the amount of anterior teeth display and the associated gingiva as an element of esthetic assessment [1]. This study showed that posed smiling was associated with slight upper lip elevation and minimum mouth opening. Therefore, the cervical thirds of the upper anterior teeth are covered by the upper lip, and the incisal parts are covered by the lower lip. Accordingly, no gingival display was recorded in all tested groups, conflicting results of Walter et al. study [28]. Besides, the present study indicated no age or gender-based significant differences in the apparent vertical anterior teeth height during posed smiling. However, aging makes central incisors less displayed, especially in the old men group. Women (young and old age) showed more visible teeth than age matched-men, agreeing with results of a previous study regarding young women [13]. The smile line height was reduced during posed smiling and was assessed as low on a posed smiling record as verified previously by Geld et al. [7].

On the contrary, in the spontaneous smile, the maxillary anterior teeth were more displayed than posed smile and were significantly related to age. Thus, younger persons showed more visible maxillary anterior teeth than old ones. This finding agrees with many other studies [3,9,10]. With age, the lips undergo several predictable changes affecting dental display. Atrophy of muscles results in the formation of the labial, nasolabial, and mental grooves and ridges. In addition, decreased lip volume, loss of elasticity and architecture of the upper lip, and lip lengthening [3,10].

No statistically significant gender differences in anterior tooth display during spontaneous smiling were recorded [17] Women displayed slightly more length of anterior maxillary teeth than men in both age groups, which agreed with previous studies [[13], [14], [15], [16], [17]]. However, the esthetic appearance of teeth displayed during smiling may be generalized over both sexes as no statistically significant gender differences were recorded [17].

In line with a previous study [14], lateral incisors are generally more visible than the central incisors or canines during posed and spontaneous smiling. The excess display of lateral incisors is due to a shorter crown with its cervical level lower than the adjacent teeth. Therefore, special care should be taken to satisfy the patient's needs for aesthetics when re-constructing maxillary lateral incisors.

Aging is associated with a reduction in maxillary smile line heights during a spontaneous smile. This reduction was significant on both lateral incisors and the right side canine regions, as observed in other previous studies [7,14,27]. The upper lip covered the cervical part of central incisors in the old age group, and the smile line heights were reduced by approximately 0.96 mm (in total) compared to 2 mm reduction in a study conducted by Geld et al. [27].

Consistent with previous studies [17,30], women exposed more gingival mucosa than men in both age groups. Men smile less frequently and intensely than women. The qualities of masculinity and femininity are essential factors in interpreting a smile [33]. Therefore, different studies have observed the importance of a smile's type and lines for improving esthetic problems [3,8,25,30].

The prevalence of the type of smile among this study subjects changed from posed to spontaneous. However, the differences were not significant except for the young women group. In posed smiles, the highest prevalence of the type of smile was low for both age groups, which contradicts previous authors who observed a predominant average type in the posed smile [3,13,30]. Nevertheless, when evaluating smile type in the spontaneous smile, excessive portions of the teeth started to display in the smile. The most presented type was the average for the young group in accordance with the previous study [8] and high for the old group.

Following a previous study [34], gummy smiles are more prevalent in young women than men. A quarter of young women presented with very high smile line compared to 5% of men. However, a minority of young subjects (5%) did not display gingiva associated with the anterior region during maximum smiling in accordance with a previous study [12], while for old, the percentage reached 20%.

To help decision about which characteristic feature is going to be the ideal representative for the Kurdish population when designing to restore missing maxillary anterior teeth, further studies are needed to evaluate the perception of the smile's esthetic appearance in a model with different present dental and gingival smile characteristic results in patients gating anterior partial or complete dental prostheses. Future studies could also be needed with the largest sample size and test the research findings on other ethnicities.

This clinical study findings reveal the necessity for increased esthetic awareness in restoring anterior maxillary teeth regarding the patient's age, especially the placement and waxing of their associated gingiva in removable prostheses or avoiding the displaying of the prosthesis-tissue junction of maxillary complete arch implant supported prostheses by rehabilitating an individual's smile line.

## 6 Conclusions

From this Kurdish sample population, the following conclusions were drawn.

1. No age or gender significant effect was seen on the display of anterior maxillary teeth in a posed smile, although women showed more of their teeth than men. While in a spontaneous smile, a significant age relation was observed.
2. No gingival display was observed in a posed smile; furthermore, aging causes the upper lip to cover more cervical portions of the central incisors.
3. Gingival display during spontaneous smiling presented significant age reduction. The smile line heights of the old group were approximately reduced by 0.96 mm than the young group. Females displayed non-significant excess gingiva than males in both age groups.
4. A low smile line (class IV) was the dominant type of smile in the posed smile. While the average smile line (class III) was the predominant type of young and the high (class II) of old in a spontaneous smile
5. Lateral incisors are more visible than adjacent teeth during both smiles.

## Ethics approval statement

The medical ethics committee of the Faculty of Dentistry, University of Sulaimani, approved this study (No:10 on September 01, 2021).

## Declaration

### Author contribution statement

Neda Mohammed AL-Kaisy: Conceived and designed the experiments; Performed the experiments; Analyzed and interpreted the data; Contributed reagents, materials, analysis tools or data; Wrote the paper.

### Data availability statement

Data will be made available on request.

### Additional information

No additional information is available for this paper.

## Funding statement

This research did not receive any specific grant from funding agencies in the public, commercial, or not-for-profit sectors.

## Declaration of competing interest

The author declare that she has no known competing financial interests or personal relationships that could have appeared to influence the work reported in this paper
